# Investigating the Short Peptidome Profile of Italian Dry-Cured Ham at Different Processing Times by High-Resolution Mass Spectrometry and Chemometrics

**DOI:** 10.3390/ijms23063193

**Published:** 2022-03-16

**Authors:** Andrea Cerrato, Sara Elsa Aita, Anna Laura Capriotti, Chiara Cavaliere, Angela Michela Immacolata Montone, Carmela Maria Montone, Aldo Laganà

**Affiliations:** 1 Department of Chemistry, Sapienza University of Rome, Piazzale Aldo Moro 5, 00185 Rome, Italy; andrea.cerrato@uniroma1.it (A.C.); saraelsa.aita@uniroma1.it (S.E.A.); chiara.cavaliere@uniroma1.it (C.C.); carmelamaria.montone@uniroma1.it (C.M.M.); aldo.lagana@uniroma1.it (A.L.); 2 Istituto Zooprofilattico Sperimentale del Mezzogiorno, Via Salute 2, Portici, 80055 Naples, Italy; angela.montone@izsmportici.it; 3 Department of Industrial Engineering, Università degli Studi di Salerno, Via Giovanni Paolo II 132, 84084 Fisciano, Italy

**Keywords:** short peptides, taste peptides, dipeptides, pyroglutamyl peptides, lactoyl dipeptides, suspect screening

## Abstract

Short peptides have been spiking interest owing to their significant contribution to the taste and functional properties of dry-cured ham. In this study, a suspect screening approach based on high-resolution mass spectrometry was employed for the comprehensive characterization of the short endogenous peptidome in dry-cured ham samples at different processing stages (14, 22, and 34 months). After careful manual spectra interpretation, a chemometric approach based on principal component analysis was employed for highlighting the differences between the three sets of samples. A total of 236 short peptide sequences was tentatively identified, including 173 natural short peptides and 63 sequences containing non-proteinogenic amino acids, the highest number ever reported for endogenous sequences in dry-cured ham. Samples in the latest processing stages presented a generally higher abundance of dipeptides, indicating residual proteolytic activity. Moreover, the several annotated modified short peptides, mainly pyroglutamination and lactoyl conjugation, allowed hypothesizing several reactions occurring over time. For the first time, several lactoyl-dipeptides were tentatively identified in dry-cured ham samples with maximum concentration in the late processing stage samples. The presented results significantly contribute to the understanding of the reaction involving short peptides that affect the sensory and functional properties of dry-cured ham.

## 1. Introduction

Dry-cured ham is a high-valuable product obtained through a traditional procedure that is typical of Mediterranean countries, e.g., the Spanish jamón Serrano and ibérico, the French jambon de Bayonne, and the Italian prosciutto crudo, and of some parts of China, like Jinhua and Xuanwei hams [1]. During the processing of curing, proteolysis is one of the main phenomena that contribute to the organoleptic characteristics of dry-cured ham, along with volatile compounds deriving from lipolysis and lipid oxidation [2]. The main proteolytic activity that occurs during ham curing is exerted by cathepsins, which act on myofibrillar and sarcoplasmic proteins [3]. Free amino acids and short peptide sequences (2–4 amino acids) have been proven to play a crucial role in the taste and flavor of dry-cured ham [4,5,6]. Hydrophobic amino acids (i.e., Leu, Val, Pro, Phe, and Trp) are known to enhance bitterness, small side-chain amino acids (i.e., Ala and Gly) and Lys promote sweet flavor [7]; and Asp and Glu are responsible for the salty and umami taste [8]. Short peptides are known to display bitterness and unpleasant smell, especially when branched-chain amino acids are present in the sequences [9], whereas glutamyl and aspartyl bound sequences are known to enhance meaty, brothy, and umami taste sensations [10].

Moreover, the biological activities of endogenous short peptides, which are independent of digestive processes and may exert their functional properties in the upper gastrointestinal tract [11], were recently studied by the group of Toldrà in dry-cured ham extracts. In 2014, four endogenous dipeptides from dry-cured ham were reported to have significant dipeptidyl peptidase IV (DPP-IV) activity [12]. Later, several dipeptides from dry-cured ham with reported angiotensin-converting enzyme (ACE) activity were annotated [13]. Recently, five dipeptides identified in Spanish ham were shown to exert significant α-glucosidase inhibitory activity [14]. 

Together with proteolytic activity, other enzymatic reactions occur on free amino acids and short peptides during curing and ripening, such as γ-glutamination, pyroglutamination, and lactoyl conjugation [15], and several non-proteinogenic amino acid derivatives have been proven to suppress bitterness and favor umami taste and kokumi [16].

Despite their crucial role in determining food taste and flavor, their wide range of biological activities, and their independence of gastrointestinal processes, short peptides have been largely neglected to date since mass spectrometric (MS) workflows borrowed from bottom-up proteomics are unsuitable for low-molecular-weight peptides that resemble metabolites rather than longer peptides [17]. In 2021, the sweet dipeptide Ala-Ala was identified and quantified in dry-cured ham by targeted MS and showed a promising ACE inhibitory activity [18]. Similarly, seven umami-enhancing dipeptides were quantified at four ripening stages by targeted MS analysis [19].

Our research group recently proposed a metabolomics-based suspect screening approach for the untargeted characterization of proteinogenic and non-proteinogenic (or more simply modified) short peptides [20]. In this paper, the endogenous short peptidome profile of Italian San Daniele ham at three different ripening stages (14, 22, and 34 months) was comprehensively studied for the first time by high-resolution MS (HRMS) and chemometrics to fill a gap in the current knowledge of dry-cured ham peptide composition. 

## 2. Results and Discussion

Compared to medium- and long-sized peptides, which have long been investigated borrowing the analytical technologies from bottom-up proteomics, short peptide sequences have been largely neglected to date [21]. Short peptides show a more remarkable resemblance to polar metabolites than longer peptides for their low molecular weights and wide range of physicochemical properties (acid-base properties and polarity). To deal with the untargeted identification of short peptide sequences, an analytical platform was set up in our laboratory based on graphitized carbon black (GCB) enrichment, suspect screening data acquisition [22], and a customized data processing workflow on Compound Discoverer software in a metabolomics-based fashion [20,23]. The suspect screening approach is based on the use of inclusion lists in the mass spectrometric method that allows bypassing the limitations of DDA mode when comprehensive lists of the analytes are available. In the case of short peptides, a dual acquisition was needed with two inclusion lists for natural and modified short peptides [20]. Moreover, the data processing workflow allowed extracting the *m/z* from the raw data files, aligning the features in the different samples, removing compounds present in the black sample, predicting the molecular formulas from the accurate masses and isotopic patterns, and associating the predicted formula to those of the short peptide sequences listed in the short peptide databases that were also employed for data acquisition. After careful manual validation of the putative peptides based on the peculiar short peptide fragmentation pathways, 173 natural short peptides and 63 peptides comprising non-proteinogenic amino acids were tentatively identified in dry-cured ham. Tables S1 and S2, for natural and modified short peptides respectively, report detailed data on the tentatively identified sequences, including retention time, proposed formula, experimental *m/z*, MS accuracy, and main diagnostic product ions. Since Leu and Ile cannot be distinguished by MS/MS (MS^3^ experiments are needed [24]), the nomenclature Xle was employed throughout the manuscript and Supplementary Materials for indicating either Leu or Ile in a peptide sequence.

### 2.1. Natural Short Peptide Profile of Dry-Cured Ham

Of the 173 annotated short peptides comprising only the 20 natural amino acids, 80 were dipeptides, 48 were tripeptides, and 45 were tetrapeptides. The most abundant amino acid was Phe (13.9%), followed by Asp (11.8%), Xle (11.6%), and Glu (10.0%). On the other hand, Met and Lys comprised only about 1% of amino acid content, and no Cys was reported in the short peptide sequences. Despite their low isoelectric point that hinders the ionization efficiency in positive ion mode, a high abundance of umami-tasting amino acids (Asp/Glu) [8] was found in the short peptide sequences, in agreement with what was reported in the literature for free amino acids [25]. 

Short peptides are released in a large amount from different muscle proteins during dry-cured ham processing by the proteolytic action of endopeptidases (cathepsins and calpains) and exopeptidases (peptidylpeptidases, aminopeptidases, and carboxypeptidases) [26]. The tentatively identified sequences were searched in those of the main sarcoplasmic (myoglobin, ubiquitin, and enzyme involved in the glycolysis) and myofibrillar (actin, myosin, titin, LIM domain and actin-binding protein, troponin T, and myozenin) proteins (Table S3). More than 80% of the annotated sequences were found in either sarcoplasmic or myofibrillar proteins (140 out of 173); this result contributed to valorizing the identification platform employed for short peptide annotation. As expected, most dipeptides were found in several protein sequences and were not informative on the primary origin of short peptide sequences. However, 32 short peptides were exclusively found in the sequences of myofibrillar proteins, whereas only 14 were uniquely found in sarcoplasmic protein sequences. 

Likewise, free amino acids, short peptides, and in particular di- and tripeptides, also have a significant role in determining the sensory properties of dry-cured ham [27]. A database search for reported sensory peptides was carried out on BIOPEP [9] and reported in Table S4. Forty-seven short peptides that were tentatively identified in dry-cured ham were found to possess at least one known sensory property, a significant result considering that the sensory peptide database comprises only 521 reported compounds. As expected, most annotated peptides (37) contributed to the bitter taste. However, several umami (15) and salty (7) taste peptides were reported due to the significant abundance of Asp and Glu in the annotated sequences. 

**Figure 1:**
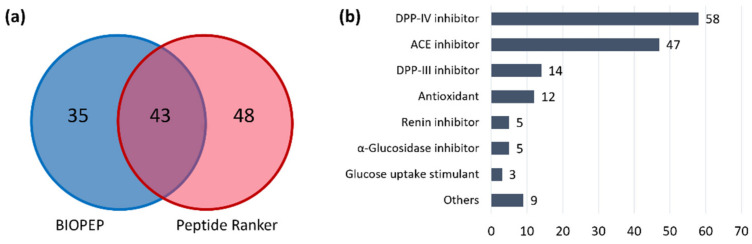
(**a**) Pie-chart reporting the short peptide sequences found in the BIOPEP-UWM database and with a score > 0.50 on PeptideRanker. (**b**) Bar chart reporting the number of peptide sequences found on BIOPEP-UWM per bioactivity.

Besides contributing to the sensorial properties of dry-cured ham, short peptides are known for their promising biological activities and for carrying out their biological potential in the upper gastrointestinal tract since they are independent of digestive properties [11]. The annotated peptides were searched in the BIOPEP-UWM database for reported bioactivities [28] and submitted to PeptideRanker, a tool to predict whether a peptide sequence is bioactive based on a neural network [29]. A total of 126 short peptides (more than 70%) was already reported on BIOPEP or had a bioactivity score > 0.50 on Peptide Ranker. More specifically, 78 annotated peptides were already reported on BIOPEP-UWM, whereas 91 had a high bioactivity score (>0.50) from PeptideRanker (43 were in common). The results are summarized in Figure 1 and reported in Table S5. 

The large number of peptides that possessed a bioactivity score > 0.50 but were not reported on BIOPEP (48) demonstrate that short peptides are very likely to carry out biological functions and that the field of short peptidomics has still much to be discovered.

The reported bioactivities are summarized in Figure 1b based on the results from the BIOPEP-UWM sequence search. Not unexpectedly, dipeptidyl peptidase IV (DPP-IV) and angiotensin-converting enzyme (ACE) inhibition were the most frequently reported activities for short peptides with 58 and 47 sequences, respectively. Fourteen annotated sequences were previously associated with DPP-III inhibitory activity, whereas twelve short peptides were demonstrated to exert antioxidant functions. Minor reported bioactivities were renin inhibition, α-glucosidase inhibition, glucose uptake stimulation, immunostimulation activity, vasoactive stimulation, anti-inflammatory activity, CaMPDE inhibition, hypolipidemic activity, anxiolytic activity, regulating ion flow activity, neurological activity, and antithrombotic activity. It is worth mentioning that the average number of reported bioactivities on BIOPEP-UWM for each of the 78 short peptides is 1.96, meaning that there is a high chance that the short bioactive peptides from dry-cured ham carry out multifunctional activities. Several dipeptides, in fact, were reported for simultaneous hypotensive (ACE inhibition) and hypoglycemic (DPP-IV inhibition) activity, hinting that short peptides could be effective nutraceuticals against metabolic syndrome [30].

### 2.2. Modified Short Peptide Profile of Dry-Cured Ham

Non-proteinogenic amino acid-containing short peptides are generated during ripening by the activity of several enzymes that are often of microbial origin [15]. For a comprehensive characterization of modified di- and tripeptides, an untargeted suspect screening approach was chosen, using a dedicated database that was employed both for data acquisition (as an inclusion list) and data processing (as a mass list) [20]. After careful short peptide identification, 63 modified short peptide sequences were tentatively identified, comprising 36 sequences containing lactic acid, 20 sequences containing pyroglutamic, 3 γ-glutamyl dipeptides, 2 peptides containing succinyl lysine, 1 methyl lysine, and 1 hydroxyproline. 

**Figure 2:**
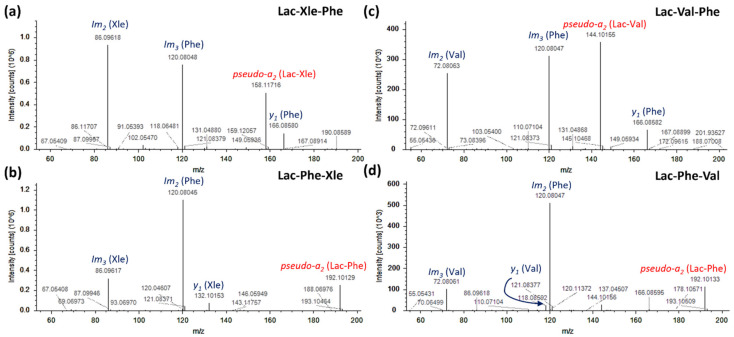
MS/MS spectra of four exemplary lactoyl-dipeptides tentatively identified in dry-cured ham samples: (**a**) Lac-Xle-Phe; (**b**) Lac-Phe-Xle; (**c**) Lac-Val-Phe; (**d**) Lac-Phe-Val.

Lactoyl conjugation of amino acids has been linked to the activity of microbial enzymes during the aging processes of meat and cheese. However, the metabolic pathways responsible for these reactions are still unknown [31]. It has been reported that lactoyl conjugates of hydrophobic and bitter amino acids (e.g., Phe, Val, Leu, Ile, Trp) carry out a bitterness suppressing activity by lowering the concentration of such amino acids during aging [32]. Not unexpectedly, lactoyl conjugates of Phe, Trp, Tyr, and Xle were tentatively identified (Table S2). However, the largest number of lactoyl-bound compounds were lactoyl-dipeptides (31 compounds), which, to the best of our knowledge, were never reported earlier in dry-cured ham or other processed food. Lactoyl-dipeptides (Lac-X2-X3) were tentatively identified according to a peculiar and consistent fragmentation pathway that involved mainly the cleavage on the peptide bond between the two natural amino acids in position 2–3 (X2–X3). This cleavage generates high abundances of pseudo-a_2_ (Lac-X2) and y_1_ (X3) ions. Moreover, iminium (im) ions of amino acids X2 and X3 confirm the presence of the two residues in the sequence. In Figure 2, four exemplary MS/MS spectra are shown. Lac-Xle-Phe shows intense ions at *m/z* 86.0962 and 120.0805 that correspond to the im ions of leucine/isoleucine and phenylalanine, respectively (im_2_ and im_3_, Figure 2a). Moreover, *m/z* 158.1172 can be attributed to a pseudo-a_2_ ion of the sequence Lac-Xle, and *m/z* 166.0858 corresponds to the usual y_1_ of phenylalanine at the C-terminus. Lac-Xle-Phe was easily distinguished from positional isomer Lac-Phe-Xle (Figure 2b) thanks to pseudo-a_2_ and y_1_ and confirmed by im_2_ and im_3_. Lac-Val-Phe (Figure 2c) and Lac-Phe-Val (Figure 2d) were annotated and distinguished using the same rationale thanks to the consistent fragmentation pathways. The large number of lactoyl-dipeptides annotated in dry-cured ham could indicate a still unknown process of bitterness suppression during ham processing.

The second-largest group of modified short peptides annotated in dry-cured ham comprised pyroglutamic acid at the N-terminus of the sequence. Pyroglutamyl amino acids and peptides impart umami taste and can be generated by cyclization of N-terminal glutamic acid and glutamine residues during heating or by the enzymatic activity of glutaminyl cyclase [33]. Pyroglutamic acid-containing short peptides were characterized by the presence of an intense im_1_ ion at *m/z* 84.0444. In general, the MS/MS spectra of these compounds were analogous to that of corresponding natural peptides with Glu or Gln after a neutral loss of H_2_O or NH_3_, respectively. 

γ-Glutamyl peptides are widespread in dairy products and are formed by the action of γ-glutamyl transferase and transpeptidase of microbial origin [34]. Those compounds are known to exert kokumi activity, meaning that they are not taste-active themselves but instead enhance the taste intensity of other compounds [16]. Compared to lactoyl conjugation and pyroglutamination, γ-glutamination is a difficult task for HRMS, since there are no differences in the fragmentation pathways compared to α-glutamination. However, γ-glutamyl amino acids are known for their increased hydrophilicity compared to the corresponding α-glutamyl conjugates. Therefore, when peptides are separated by RP, γ-glutamyl conjugates elute earlier than the corresponding α-bound isomers [35]. As such, when two isomers were annotated, retention times (RT) were used for their discrimination, as in the case of α-Glu-Phe and γ-Glu-Phe, which eluted at RT 8.7 and 7.8, respectively. Only three γ-glutamyl amino acids were annotated, but the actual number of these sequences among the tentatively identified ones with N-terminal glutamic acid is likely larger. In Figure S1, the chromatographic peaks of isomeric glutamyl-amino acids are reported.

Of the three minor modified amino acids reported (succinyl-lysine, methyl-lysine, and hydroxyproline), succinylation has also raised interest for a possible role in enhancing the umami taste. 

### 2.3. Principal Component Analysis of the Short Peptidome Profile of Dry-Cured Ham

**Figure 3:**
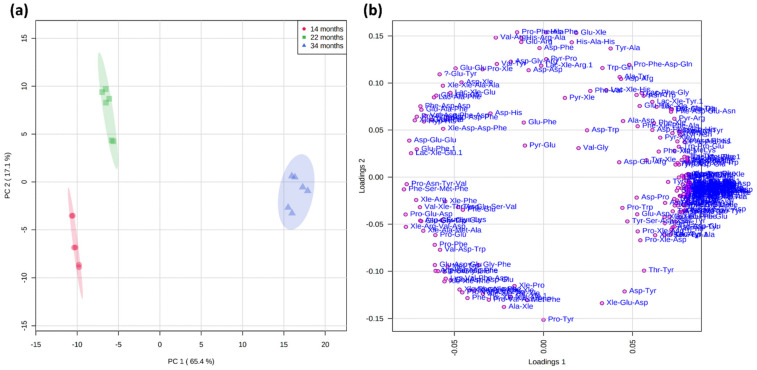
PCA modeling (PC1 vs. PC2) of dry-cured ham samples at the three different processing stages T1–T3 (14, 22, and 34 months) based on the short peptide datasets. (**a**) scores plot; (**b**) loadings plot.

For the evaluation of the effect of aging on the short peptidome profile, an unsupervised chemometric approach was employed based on the principal component analysis (PCA). The data matrix obtained from Compound Discoverer after alignment of the different runs and short peptide annotation was submitted to MetaboAnalyst, a freeware for metabolomics and chemometrics analyses [36]. The PCA is often employed for exploratory data analysis, and is based on the least square approximation of the data that are projected on a reduced set of latent variables (principal components, PC) that describe the largest variability of the experimental datasets [37]. Before the statistical analysis, a normalization step based on autoscaling, i.e., mean-centering and dividing by the standard deviation of each variable, was performed so that each peptide had a similar contribution (the results are shown in Figure S2). Information on the relations among the samples is displayed in the scores plot along the principal components, whereas the interpretation of the compounds can be investigated on the loadings plot. The 236 annotated short peptides (173 natural and 63 modified sequences) were used as variables. In Figure 3, the PCA modeling of dry-cured ham at the three processing stages (14, 22, and 34 months) is shown. The contribution of the PC1 was 65.4%, while PC2 contributed for 17.1% of the total variance (when combined, PC1 and PC2 constituted more than 80% of the total variance). Early processing stages T1–T2 (14 and 22 months, respectively) are clearly discriminated from the late processing stage samples (T3, 34 months) alongside PC1, with negative values for T1–T2 and positive values for T3. Therefore, a trend can be observed, with the most negative values for T1. Conversely, T1 and T2 are discriminated against alongside PC2, with negative values for the former and positive values for the latter. PC3 and PC4 contributed for only 4.7% and 4.4% of the total variance, respectively, and the resulting scores plot against PC1 are no anymore informative (Figures S3 and S4). As such, the trend observed in the PC1 vs. PC2 scores plot alongside PC1 is the only visible information when PC3/PC4 is shown. The substantial difference between T2 and T3 compared to the two early processing sets of samples (T1–T2) could be partially explained by the larger difference (12 months) between the second and third sets of samples. Those results demonstrate that the short peptidome profile of dry-cured ham could be efficiently employed for discriminating dry-cured ham samples at different processing stages.

By inspecting the loadings plot, it appears that most peptide sequences had higher concentrations in the 34-month samples, a result that appears in agreement with the activity of exo- and endopeptidases during aging. However, it has been demonstrated that cathepsins show much lower activity in comparison with that initially measured in fresh meat already after 14–15 months of processing [3,38]. Therefore, other proteolytic and non-proteolytic enzymes were possibly responsible for these variations in late processing stages. 

The inspection of the loadings plot allowed a better understanding of the reactions that transformed the short peptidome over time. Several dipeptides showed a higher area at T2 compared to T1 (Figure S5), indicating residual proteolytic activity of cathepsins or other enzymes. These results are supported by several tetrapeptides possessing higher area at T1 than T2–T3 (Figure S6). As mentioned, however, most variations were found between T2 and T3. In particular, non-proteinogenic amino acid-containing peptides, i.e., lactoyl amino acids and dipeptides, pyroglutamyl amino acids and dipeptides, and γ-glutamyl amino acids, showed a critical increase from T2 to T3, demonstrating an intense enzymatic activity in the late stages of processing by a series of enzymes that are likely of microbial origin. In Figure S7, the abundances of three exemplary modified peptides in the three sets of samples are shown. 

**Figure 4:**
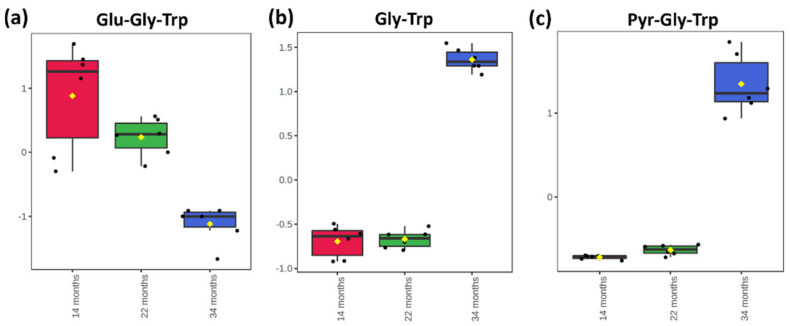
Box and whisker plots showing the abundance of the short peptide with an N-terminal glutamic acid Glu-Gly-Trp (**a**), and two hypothetical derivatives Gly-Trp (**b**) and Pyr-Gly-Trp (**c**) in the three sets of samples.

The opportunity to obtain a comprehensive short peptidome identification has allowed following the fate of single peptides over time and, eventually, hypothesizing the reaction mechanisms. Glutamic acid and glutamine residues at the N-terminus are prone to several reactions, including enzymatic and non-enzymatic pyroglutamination [39]. Moreover, a residual proteolytic activity could result in further breakdown of tri- and tetrapeptides. In Figure 4, the intensity trend of Glu-Gly-Trp is shown alongside two possible derivatives Gly-Trp and Pyr-Gly-Trp. Based on these results, it is possible to hypothesize a competition between proteolytic reactions and glutamic acid dehydration. The trends of other short peptides identified in dry-cured ham with N-terminal glutamic acid residues are shown in Figure S8. Among the six exemplary peptides, it is worth mentioning that longer sequences tend to decrease earlier (from T1 to T2) than shorter peptides, which instead increase from T1 to T2 before dramatically decreasing at T3. These trends corroborated the hypothesis of residual proteolytic activity from T1 to T2 that generates di- and tripeptides from longer sequences. 

**Figure 5:**
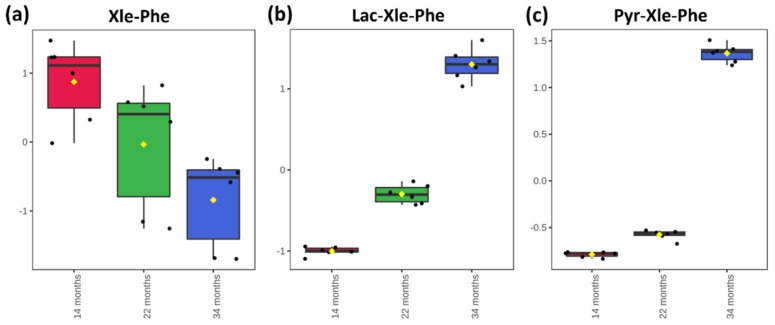
Box and whisker plots showing the abundance of bitter taste dipeptide Xle-Phe (**a**), and two hypothetical derivatives Lac-Xle-Phe (**b**) and Pyr-Xle-Phe (**c**) in the three sets of samples.

Dipeptides contribute to a great extent to the characteristic taste of dry-cured ham. In particular, several dipeptides comprising aromatic and branched-chain amino acids, such as phenylalanine, tyrosine, tryptophan, leucine, isoleucine, and valine, have a significant role in the bitter taste of dry-cured ham [8]. In a recent paper by Gallego et al. [19], seven *umami* and bitter taste dipeptides were quantified by LC-MS/MS in dry-cured ham samples at four processing stages (6, 12, 18, and 24 months). While an increase was found for umami taste dipeptides (including Glu-Glu, for which an increase from T1 to T2 was also found in the present study), bitter taste Val-Gly was found to decrease. The authors correctly attributed the result to further breakdown into amino acids. The identification of lactoyl dipeptides in the present study furnished a new possible explanation for the decrease of bitter taste dipeptides over time. It is known, in fact, that lactoyl conjugation affects mainly hydrophobic residues [31] and, as a matter of fact, of the 36 annotated lactoyl bound sequences, 14 presented a Lac-Phe conjugation, 12 presented a Lac-Xle conjugation, 3 a Lac-Trp conjugation, and 2 each presented Lac-Tyr and Lac-Val conjugations. In Figure 5, the decreasing trend from T1 to T3 of bitter taste dipeptide Xle-Phe is shown, along with the increasing abundances of Lac-Xle-Phe and Pyr-Xle-Phe, which suppress the bitter taste and contribute to the umami taste of processed dry-cured ham.

The large number of lactoyl-dipeptides that were tentatively identified in the dry-cured ham samples at late processing stages could indicate a still unknown mechanism of bitterness suppression and umami promotion.

## 3. Materials and Methods

### 3.1. Chemicals and Sample Collection

Dry-cured ham samples at three different curing stages (14, 21, and 34 months) were kindly provided by Consorzio del Prosciutto di San Daniele (San Daniele del Friuli, Italy). All chemicals and reagents were purchased from Merck (St. Louis, MO, USA), unless otherwise stated. Optima LC-MS grade water and acetonitrile (ACN) were purchased from Thermo Fisher Scientific (Waltham, MA, USA). Ultrapure water was prepared by an Arium 611 VF system from Sartorius (Göttingen, Germany). Cartridges packed with 500 mg Carbograph 4 were supplied by Lara S.R.L. (Lara S.r.l., Formello, Italy).

### 3.2. Short Peptide Extraction

Dry-cured ham samples were first homogenized by an Imetec HM3 (Tenacta Group SpA, Azzano San Paolo, Italy). One g of each sample was extracted with 10 mL Tris-HCl buffer at 100 mmol L^−1^ (pH 8) by alternating vortexing and sonication for 30 min following centrifugation at 4 °C and 8000 rpm for 15 min. Later, proteins were precipitated overnight by adding ACN (30 mL, 1:3 ratio) following centrifugation at 4 °C and 8000 rpm for 15 min to remove the protein pellet. ACN was later removed by evaporation using an IKA RV 8 rotary evaporator (IKA-Werke GmbH & Co. KG, Staufen, Germany). Fat constituents were then removed by liquid–liquid extraction with 10 mL of hexane. Finally, the aqueous extracts (10 mL) were acidified at pH 2 with TFA for subsequent GCB purification.

### 3.3. Short Peptide Purification on GCB

Short peptides were purified onto Carbograph 4 cartridges using a previously optimized protocol for short peptides [23]. Briefly, the cartridge was washed and activated with 10 mL of 0.1 mol L^−1^ HCl and conditioned with 10 mL of 20 mmol L^−1^ TFA before sample loading. The cartridge was then washed with 5 mL of 20 mmol L^−1^ TFA. The eluates were dried in a thermostatic bath at 25 °C under nitrogen flow. The residue was finally reconstituted in 1 mL of water for subsequent liquid chromatography coupled to HRMS (LC–HRMS) analysis.

### 3.4. Liquid Chromatography-Mass Spectrometry and Short Peptide Identification

Natural and modified short peptides were analyzed by LC–HRMS in suspect screening fashion, as previously described [20] using a Vanquish binary pump coupled to a hybrid quadrupole-Orbitrap Q Exactive mass spectrometer (Thermo Fisher Scientific) through a heated electrospray source. Samples were separated by a Kinetex XB-C18 (100 × 2.1 mm, particle size 2.6 μm, Phenomenex, Torrance, CA, USA) operated at 40 °C. Spectra were acquired in the positive ion mode in the range *m/z* 150–750 with a resolution (full width at half maximum, FWHM, *m/z* 200) of 70,000. For each sample, two individual runs were acquired for natural and modified short peptides using two dedicated inclusion lists containing the exact *m/z* of the protonated ions of all unique short peptide mass. The inclusion lists were prepared using MatLab R2018, as previously described [20]. The acquisition of the higher collisional dissociation (HCD) MS/MS spectra was performed using the top 5 data-dependent acquisition (DDA) mode at 35% normalized collision energy and 35,000 (FWHM, *m/z* 200) resolution. All samples were run in triplicate.

The identification of the short endogenous peptidome was achieved thanks to a dedicated data processing workflow implemented on Compound Discoverer 3.1 (Thermo Fisher Scientific) by our research group [20]. The strategy allowed to extract the *m/z* from the RAW data files, aligning the runs and removing blank signals and masses not associated with at least one MS/MS spectrum. Moreover, the workflow allowed to filter out all masses not present in the mass list (the same employed for short peptide data acquisition). The identification of the short sequences was achieved after manual interpretation of the MS/MS spectra aided by the match of the in silico spectra generated by mMass [40]. The tentatively identified short peptides were searched on the BIOPEP–UWM database [28] for reported biological activities. Moreover, Peptide Ranker [29] was used to predict the bioactivity of the annotated sequences.

### 3.5. Statistical Analysis

MetaboAnalyst 5.0 was employed for statistical analysis of the short peptide datasets [36]. The data matrix was submitted as a text file that was prepared according to the specific indications that are furnished by the developers. The interquartile range (IQR) was selected for data filtering, whereas the autoscaling algorithm was selected for data scaling. An unsupervised chemometric approach based on the principal component analysis was chosen for the evaluation of the four sets of samples.

## 4. Conclusions

Short peptides are a tricky class of peptides to investigate comprehensively; yet, their role in determining food taste, flavor, and functional properties (mainly processed food) is significant. In this study, a comprehensive short peptidome characterization of dry-cured ham was achieved, thanks to a dedicated analytical platform covering all phases of untargeted HRMS analysis (sample preparation, data acquisition, and data analysis). Among the numerous annotated sequences, many of these possessed taste properties and were short bioactive peptides. Moreover, the large number of short peptides allowed hypothesizing many of the reactions occurring to peptides during processing that affect the short peptidome and, subsequently, the taste and functional properties of dry-cured ham. The omics-based approach allowed to annotate, for the first time, lactoyl-dipeptides, a neglected class of short peptide derivatives that could play a significant role in the bitterness depression and umami promotion of dry-cured ham during processing. 

## Supplementary Materials

The following supporting information can be downloaded at: https://www.mdpi.com/article/10.3390/ijms23063193/s1, Figure S1. Chromatographic peaks of annotated α and γ-glutamyl amino acids in dry-cured ham samples. Retention time was employed for discriminating the two isomers; Figure S2. Summary of the normalization of the short peptides performed by autoscaling of each variable. The boxplots show 50 features/samples due to space limitation; the density plots are based on all data; Figure S3. PCA modeling (PC1 vs. PC3, scores plot) of dry-cured ham samples at the three different processing stages (14, 22, and 34 months) based on the short peptide datasets; Figure S4. PCA modeling (PC1 vs. PC4, scores plot) of dry-cured ham samples at the three different processing stages (14, 22, and 34 months) based on the short peptide datasets; Figure S5. Box and whisker plots showing the abundances of three exemplary dipeptides Asn-Trp (a), His-Phe (b), and Trp-Gln (c) that had a significant increase from T1 to T2; Figure S6. Box and whisker plots showing the abundances of three exemplary tetrapeptides Glu-Asp-Asp-Glu (a), Phe-Thr-Xle-Xle (b), and Xle-Phe-Gly-Xle (c) that had a significant decrease from T1 to T2; Figure S7. Box and whisker plots showing the abundances of three exemplary non-proteinogenic amino acid-containing peptides Pyr-Glu-Tyr (a), Lac-Phe-Xle (b), and γ-Glu-Trp (c) that had a significant increase from T2 to T3; Figure S8. Box and whisker plots showing the abundances of short peptides with an N-terminal glutamic acid Glu-Asp-Asp-Glu (a), Glu-Asp-Glu (b), Glu-Ala-Phe (c), Glu-Glu-Glu (d), Glu-Glu (e), and α-Glu-Phe that had a significant decrease from T1/T2 to T3; Table S1. Retention times (RT, min), proposed formulas, experimental *m*/*z*, accuracy (Δ, ppm), adducts, 1-letter nomenclature, and diagnostic product ions (*m*/*z*) of the 173 tentatively identified natural short peptides in dry-cured ham; Table S2. Retention times (RT, min), proposed formulas, experimental m/z, accuracy (Δ, ppm), adducts, and diagnostic product ions (*m*/*z*) of the 63 tentatively identified modified short peptides in dry-cured ham; Table S3. Results of the manual search of the annotated short peptides in the sequences of the major sarcoplasmic and myofibrillar protein of Sus Scrofa (Source UNIPROT); Table S4. Results of the database search of the annotated natural short peptides in dry-cured ham on the Sensory peptides and amino acid database of BIOPEP-UWM; Table S5. Results of the database search of the annotated natural short peptides in dry-cured ham on the Bioactive peptides database of BIOPEP-UWM.

## References

[1] Toldrá F., Gallego M., Reig M., Aristoy M.-C., Mora L. (2020). Bioactive peptides generated in the processing of dry-cured ham. Food Chem..

[2] Toldrá F., Flores M. (1998). The Role of Muscle Proteases and Lipases in Flavor Development During the Processing of Dry-Cured Ham. Crit. Rev. Food Sci. Nutr..

[3] Toldrá F., Rico E., Flores J. (1993). Cathepsin B, D, H and L activities in the processing of dry-cured ham. J. Sci. Food Agric..

[4] Jurado Á., García C., Timón M.L., Carrapiso A.I. (2007). Effect of ripening time and rearing system on amino acid-related flavour compounds of Iberian ham. Meat Sci..

[5] Sentandreu M.A., Stoeva S., Aristoy M.C., Laib K., Voelter W., Toldra E. (2003). Identification of Small Peptides Generated in Spanish Dry-cured Ham. J. Food Sci..

[6] Sforza S., Pigazzani A., Motti M., Porta C., Virgili R., Galaverna G., Dossena A., Marchelli R. (2001). Oligopeptides and free amino acids in Parma hams of known cathepsin B activity. Food Chem..

[7] Stadnik J., Dolatowski Z.J. (2015). Free Amino Acids and Biogenic Amines Content during Ageing of Dry-cured Pork Loins Inoculated with Lactobacillus casei ŁOCK 0900 Probiotic Strain. Food Sci. Technol. Res..

[8] Kęska P., Stadnik J. (2017). Taste-active peptides and amino acids of pork meat as components of dry-cured meat products: An in-silico study. J. Sens. Stud..

[9] Iwaniak A., Minkiewicz P., Darewicz M., Sieniawski K., Starowicz P. (2016). BIOPEP database of sensory peptides and amino acids. Food Res. Int..

[10] Van den Oord A.H.A., van Wassenaar P.D. (1997). Umami peptides: Assessment of their alleged taste properties. Z. Leb. Forsch. A.

[11] Webb K.E., Matthews J.C., DiRienzo D.B. (1992). Peptide absorption: A review of current concepts and future perspectives. J. Anim. Sci..

[12] Gallego M., Aristoy M.-C., Toldrá F. (2014). Dipeptidyl peptidase IV inhibitory peptides generated in Spanish dry-cured ham. Meat Sci..

[13] Gallego M., Mora L., Hayes M., Reig M., Toldrá F. (2019). Peptides with Potential Cardioprotective Effects Derived from Dry-Cured Ham Byproducts. J. Agric. Food Chem..

[14] Mora L., González-Rogel D., Heres A., Toldrá F. (2020). Iberian dry-cured ham as a potential source of α-glucosidase-inhibitory peptides. J. Funct. Foods.

[15] Paolella S., Prandi B., Falavigna C., Buhler S., Dossena A., Sforza S., Galaverna G. (2018). Occurrence of non-proteolytic amino acyl derivatives in dry-cured ham. Food Res. Int..

[16] Zhao C.J., Schieber A., Gänzle M.G. (2016). Formation of taste-active amino acids, amino acid derivatives and peptides in food fermentations—A review. Food Res. Int..

[17] Peng J., Zhang H., Niu H., Wu R. (2020). Peptidomic analyses: The progress in enrichment and identification of endogenous peptides. TrAC Trends Anal. Chem..

[18] Heres A., Yokoyama I., Gallego M., Toldrá F., Arihara K., Mora L. (2021). Antihypertensive potential of sweet Ala-Ala dipeptide and its quantitation in dry-cured ham at different processing conditions. J. Funct. Foods.

[19] Gallego M., Toldrá F., Mora L. (2022). Quantification and in silico analysis of taste dipeptides generated during dry-cured ham processing. Food Chem..

[20] Cerrato A., Aita S.E., Capriotti A.L., Cavaliere C., Montone C.M., Laganà A., Piovesana S. (2020). A new opening for the tricky untargeted investigation of natural and modified short peptides. Talanta.

[21] Fricker L.D. (2015). Limitations of Mass Spectrometry-Based Peptidomic Approaches. J. Am. Soc. Mass Spectrom..

[22] Montone C.M., Capriotti A.L., Cerrato A., Antonelli M., La Barbera G., Piovesana S., Laganà A., Cavaliere C. (2019). Identification of bioactive short peptides in cow milk by high-performance liquid chromatography on C18 and porous graphitic carbon coupled to high-resolution mass spectrometry. Anal. Bioanal. Chem..

[23] Piovesana S., Capriotti A.L., Cerrato A., Crescenzi C., La Barbera G., Laganà A., Montone C.M., Cavaliere C. (2019). Graphitized Carbon Black Enrichment and UHPLC-MS/MS Allow to Meet the Challenge of Small Chain Peptidomics in Urine. Anal. Chem..

[24] Xiao Y., Vecchi M.M., Wen D. (2016). Distinguishing between Leucine and Isoleucine by Integrated LC–MS Analysis Using an Orbitrap Fusion Mass Spectrometer. Anal. Chem..

[25] Virgili R., Parolari G., Bordini C.S., Schivazappa C., Cornet M., Monin G. (1999). Free Amino Acids and Dipeptides in Dry-Cured Ham. J. Muscle Foods.

[26] Toldra F. (1998). Proteolysis and lipolysis in flavour development of dry-cured meat products. Meat Sci..

[27] Gallego M., Mora L., Toldrá F. (2019). The relevance of dipeptides and tripeptides in the bioactivity and taste of dry-cured ham. Food Prod. Process. Nutr..

[28] Minkiewicz P., Iwaniak A., Darewicz M. (2019). BIOPEP-UWM Database of Bioactive Peptides: Current Opportunities. Int. J. Mol. Sci..

[29] Mooney C., Haslam N.J., Pollastri G., Shields D.C. (2012). Towards the Improved Discovery and Design of Functional Peptides: Common Features of Diverse Classes Permit Generalized Prediction of Bioactivity. PLoS ONE.

[30] Craik D.J., Fairlie D.P., Liras S., Price D. (2013). The Future of Peptide-based Drugs. Chem. Biol. Drug Des..

[31] Sgarbi E., Lazzi C., Iacopino L., Bottesini C., Lambertini F., Sforza S., Gatti M. (2013). Microbial origin of non proteolytic aminoacyl derivatives in long ripened cheeses. Food Microbiol..

[32] Sforza S., Cavatorta V., Galaverna G., Dossena A., Marchelli R. (2009). Accumulation of non-proteolytic aminoacyl derivatives in Parmigiano-Reggiano cheese during ripening. Int. Dairy J..

[33] Schilling S., Stenzel I., von Bohlen A., Wermann M., Schulz K., Demuth H.-U., Wasternack C. (2007). Isolation and characterization of the glutaminyl cyclases from Solanum tuberosum and Arabidopsis thaliana: Implications for physiological functions. Biol. Chem..

[34] Toelstede S., Hofmann T. (2009). Kokumi-Active Glutamyl Peptides in Cheeses and Their Biogeneration by Penicillium roquefortii. J. Agric. Food Chem..

[35] Szókán G., Kelemen G., Török A. (1986). High-performance liquid chromatography of isopeptides. J. Chromatogr. A.

[36] Xia J., Wishart D.S. (2011). Metabolomic Data Processing, Analysis, and Interpretation Using MetaboAnalyst. Current Protocols in Bioinformatics.

[37] Bro R., Smilde A.K. (2014). Principal component analysis. Anal. Methods.

[38] Piasentier E., Pizzutti N., Lippe G. (2021). The Influence of the Type of Dry-Cured Italian PDO Ham on Cathepsin B Activity Trend during Processing. Foods.

[39] Gazme B., Boachie R.T., Tsopmo A., Udenigwe C.C. (2019). Occurrence, properties and biological significance of pyroglutamyl peptides derived from different food sources. Food Sci. Hum. Wellness.

[40] Strohalm M., Kavan D., Novák P., Volný M., Havlíček V. (2010). mMass 3: A Cross-Platform Software Environment for Precise Analysis of Mass Spectrometric Data. Anal. Chem..

