# Supplementary material for: Investigating the Short Peptidome Profile of Italian Dry-Cured Ham at Different Processing Times by High-Resolution Mass Spectrometry and Chemometrics
Source: Int J Mol Sci. 2022 Mar 16;23(6):3193. doi: 10.3390/ijms23063193 (PMC8951069; doi:10.3390/ijms23063193)
Supplement: Supplementary file 1 [file ijms-23-03193-s001.zip › Supplementary Material.pdf]

Supplementary Material

Investigating the short peptidome profile of Italian dry-cured ham at different processing times by high-resolution mass spectrometry and chemometrics

Andrea Cerrato [1], Sara Elsa Aita [1], Anna Laura Capriotti [1,*], Chiara Cavaliere [1], Angela Michela Immacolata Montone [2,3], Carmela Maria Montone [1], and Aldo Laganà [1]

1 Department of Chemistry, Sapienza University of Rome, Piazzale Aldo Moro 5, 00185, Rome, Italy; andrea.cerrato@uniroma1.it (A.C.); saraelsa.aita@uniroma1.it (S.E.A.); chiara.cavaliere@uniroma1.it (C.C.); carmelamaria.montone@uniroma1.it (C.M.M.); aldo.lagana@uniroma1.it (A.L.);

2 Istituto Zooprofilattico Sperimentale del Mezzogiorno, Via Salute 2, Portici, 80055 Naples, Italy; angela.montone@izsmportici.it (A.M.I.M.)

3 Department of Industrial Engineering, Università degli Studi di Salerno, Via Giovanni Paolo II 132, 84084 Fisciano, Italy

* Correspondence: annalaura.capriotti@uniroma1.it (A.L.C.); Tel.: (optional; include country code; if there are multiple corresponding authors, add author initials)

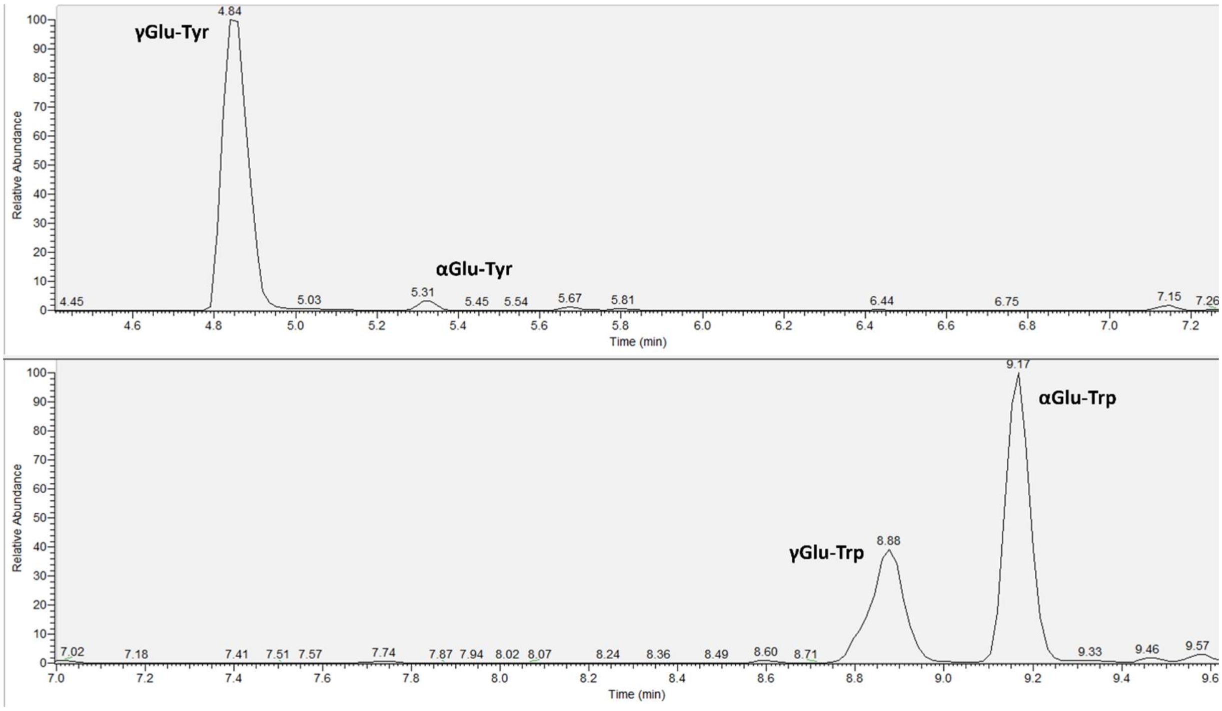

Figure S1. Chromatographic peaks of annotated α and γ-glutamyl amino acids in dry-cured ham samples. Retention time was employed for discriminating the two isomers.

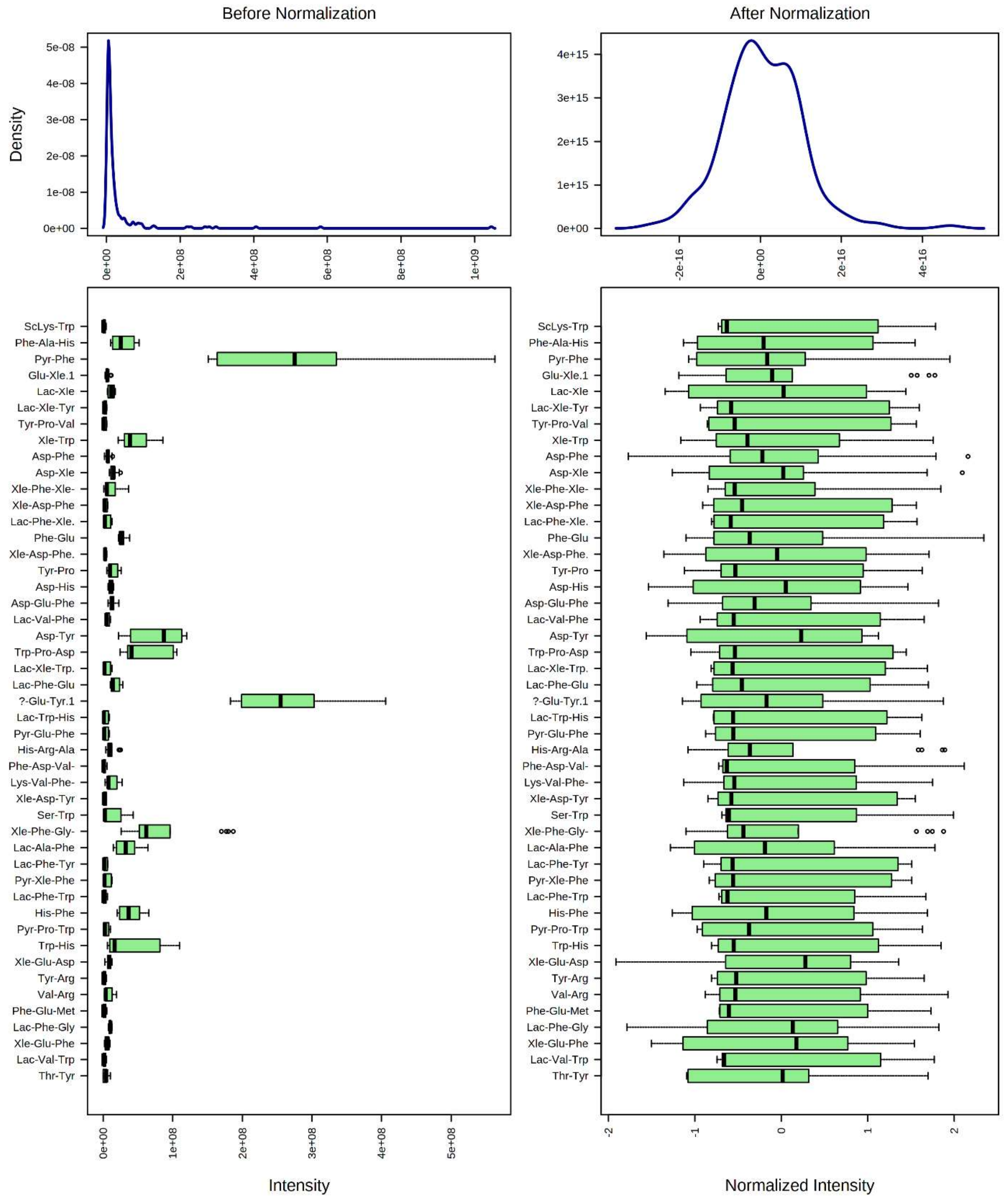

Figure S2. Summary of the normalization of the short peptides performed by autoscaling of each variable. The boxplots show 50 features/samples due to space limitation; the density plots are based on all data.

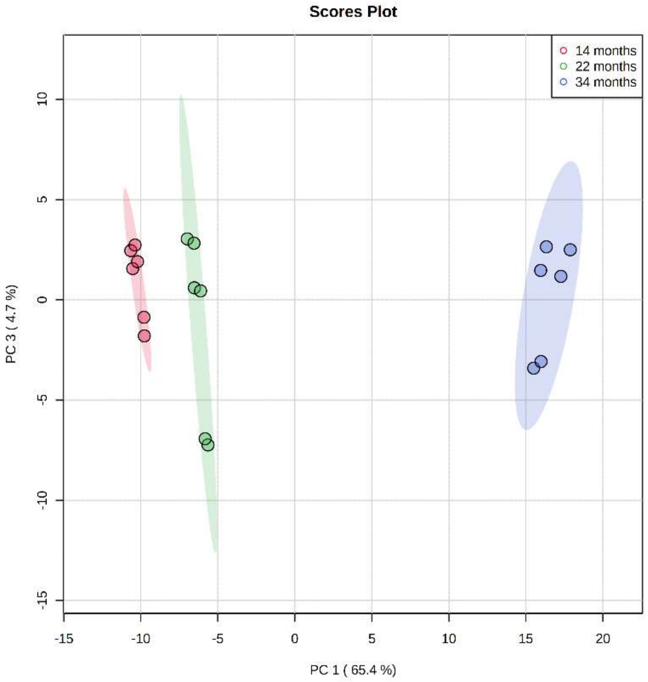

Figure S3. PCA modeling (PC1 vs PC3, scores plot) of dry-cured ham samples at the three different processing stages (14, 22, and 34 months) based on the short peptide datasets.

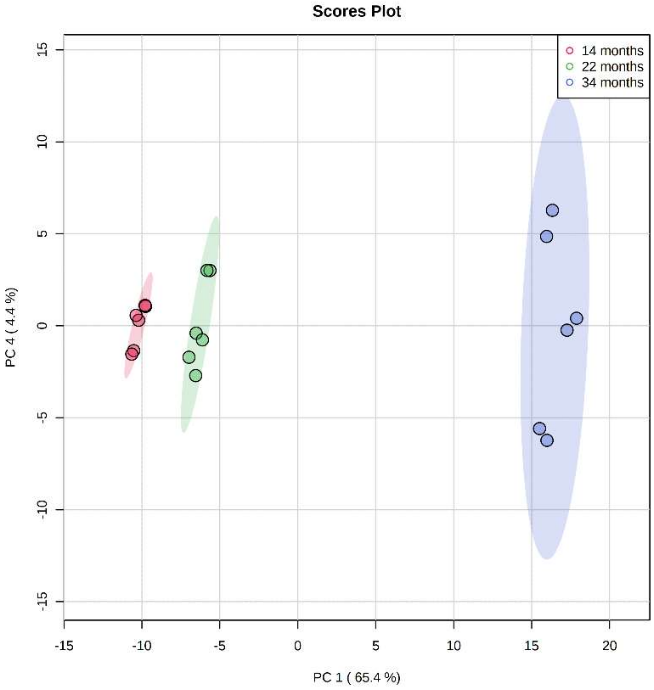

Figure S4. PCA modeling (PC1 vs PC4, scores plot) of dry-cured ham samples at the three different processing stages (14, 22, and 34 months) based on the short peptide datasets.

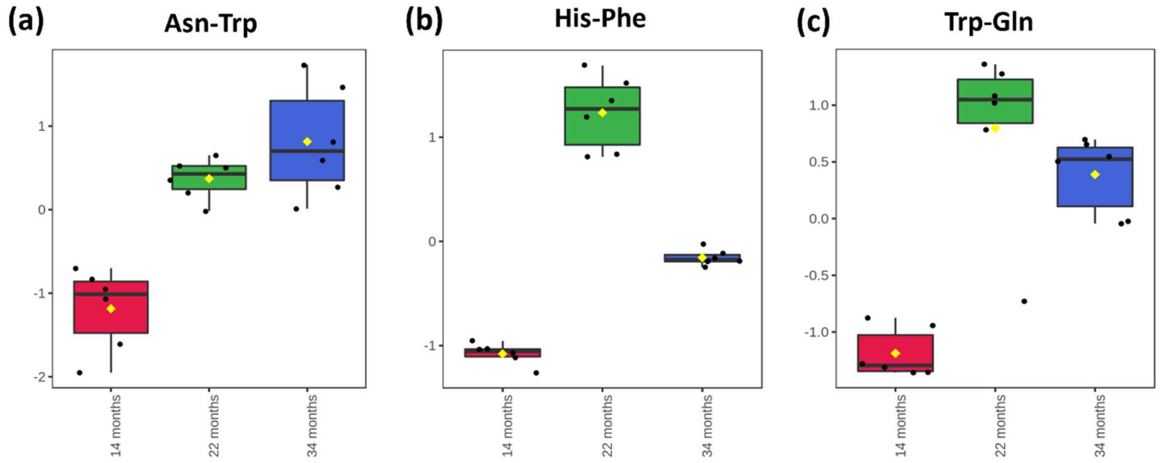

Figure S5. Box and whisker plots showing the abundances of three exemplary dipeptides Asn-Trp (a), His-Phe (b), and Trp-Gln (c) that had a significant increase from T1 to T2.

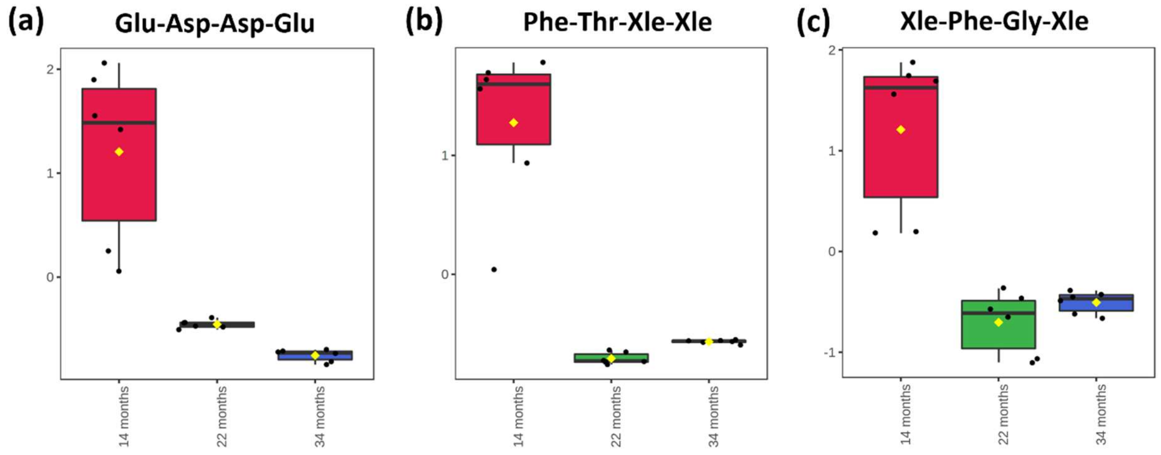

Figure S6. Box and whisker plots showing the abundances of three exemplary tetrapeptides Glu-Asp-Asp-Glu (a), Phe-Thr-Xle-Xle (b), and Xle-Phe-Gly-Xle (c) that had a significant decrease from T1 to T2.

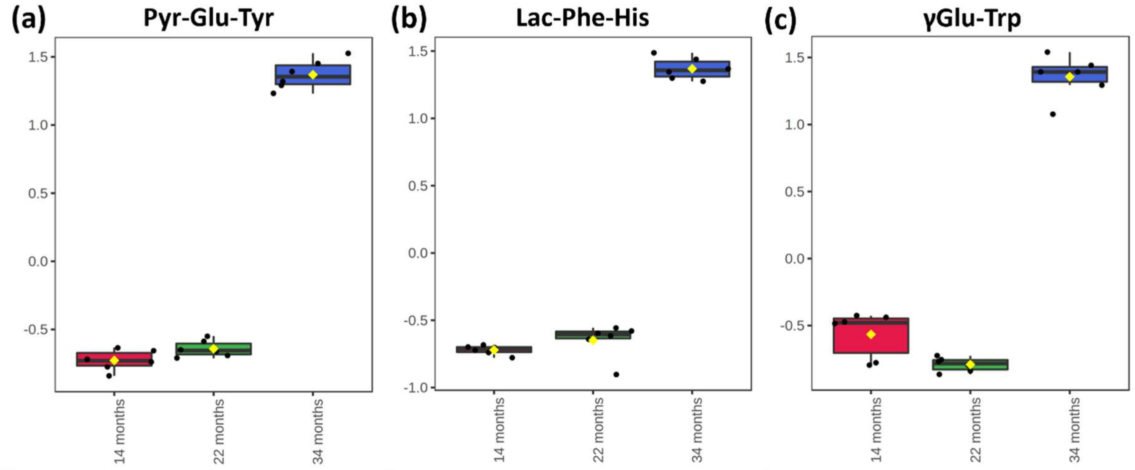

Figure S7. Box and whisker plots showing the abundances of three exemplary non-proteinogenic amino acid-containing peptides Pyr-Glu-Tyr (a), Lac-Phe-Xle (b), and γ-Glu-Trp (c) that had a significant increase from T2 to T3.

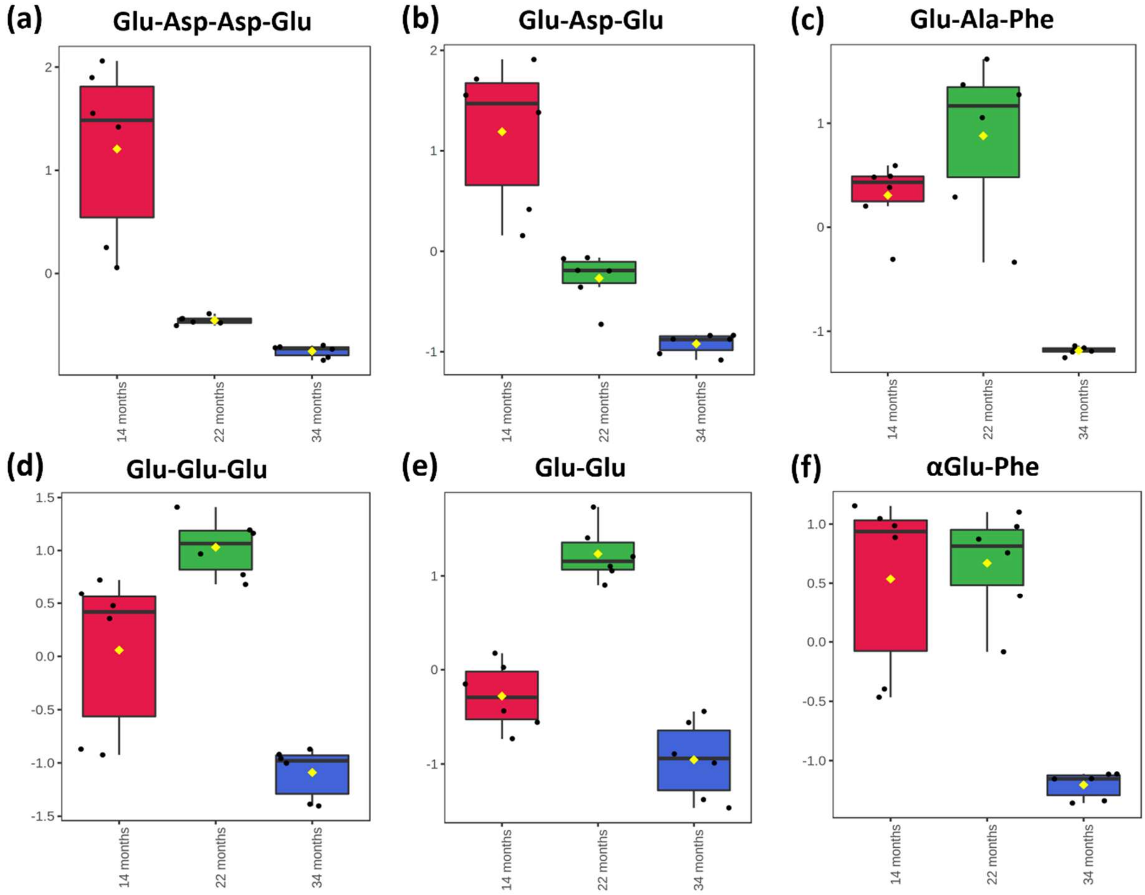

Figure S8. Box and whisker plots showing the abundances of short peptides with an N-terminal glutamic acid Glu-Asp-Asp-Glu (a), Glu-Asp-Glu (b), Glu-Ala-Phe (c), Glu-Glu-Glu (d), Glu-Glu (e), and *α*-Glu-Phe that had a significant decrease from T1/T2 to T3.
